# Vasoactive Intestinal Peptide-Secreting Pancreatic Neuroendocrine Tumor: A Case Report

**DOI:** 10.7759/cureus.22819

**Published:** 2022-03-03

**Authors:** Sritharan Thivacaren, Navaneethakrishnan Suganthan, Vathulan Sujanitha, Vengadasalam Sutharshan

**Affiliations:** 1 Internal Medicine, Teaching Hospital Jaffna, Jaffna, LKA; 2 Medicine, University of Jaffna, Jaffna, LKA; 3 Surgery, Teaching Hospital Jaffna, Jaffna, LKA

**Keywords:** chronic diarrhea, distal pancreatectomy, octreotide, hypokalemia, vasoactive intestinal peptide tumor

## Abstract

A 36-year-old female with chronic watery diarrhea and persistent hypokalemia for more than eight months duration eventually being diagnosed as vasoactive intestinal peptide tumor (VIPoma) clinically and histologically is presented here. The patient achieved complete recovery after starting octreotide, a somatostatin analog. She underwent a distal pancreatectomy along with the removal of the tumor at Teaching Hospital Jaffna for the permanent cure.

## Introduction

Vasoactive intestinal peptide tumor (VIPoma), also known as Verner-Morrison syndrome, is a rare type of neuroendocrine tumor. It was first reported in the literature in 1958 by Verner and Morrison [1]. It is also called watery diarrhea, hypokalemia, and achlorhydria (WDHA) syndrome. Any patient with chronic watery diarrhea and hypokalemia should be evaluated for VIPoma, as early diagnosis is crucial to improve outcomes.

## Case presentation

A 36-year-old Sri Lankan female, a mother of three children, presented with a history of chronic watery diarrhea for eight months duration. It was a secretory type of large voluminous watery diarrhea with a frequency of eight times per day. It was not mixed with blood or mucous and was not associated with abdominal pain. She had nocturnal diarrhea, and it was persisted despite fasting. There was no associated fever, facial flushing, vomiting, steatorrhea, alternating constipation, or fecal incontinence. Her past medical history was significant for bronchial asthma, which had been well controlled with inhaled corticosteroids. Other than chronic diarrhea, no other symptoms suggestive of hyperthyroidism were noted. She had an unintentional weight loss of 15 kg over a period of the last eight months due to persistent diarrhea. She has not been on any regular medication other than the inhaled steroid.

On examination, she was afebrile and not pale or icteric, and her body mass index was 23 kg/m^2^. Her pulse rate was 82 beats per minute, and her blood pressure was 110/70 mmHg. She had no signs of malnutrition, viz., glossitis, easy bruising, skin rashes, and peripheral edema. Examination of her abdomen revealed no organomegaly or any other masses or free fluid. The rest of the clinical examination was unremarkable.

**Figure 1:**
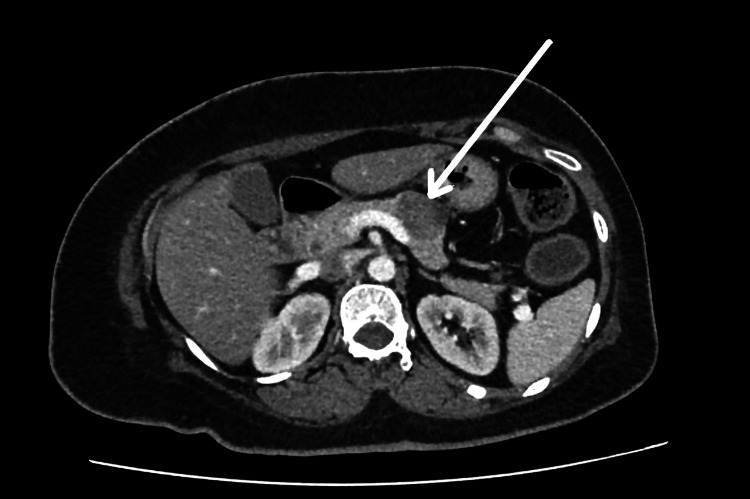
Contrast-enhanced computed tomography of the chest, abdomen, and pelvis revealing peripherally enhancing well-defined local lesion in the body of the pancreas measuring 3.2 × 2.7 × 2.8 cm with a small cystic component

Her initial investigations are shown in Table 1. The most specific investigation finding was resistant hypokalemia, which ranges between 2 and 2.5 mmol/L despite intravenous and oral potassium supplements. Her stool osmolar studies showed sodium of 83 mmol/L and potassium of 56.17 mmol/L with an osmolar gap of 12 mOsmol/kg (secretory diarrhea is usually less than 50 mOsmol/L). Her ABG revealed a pH of 7.32 and bicarbonate 12.6 mmol/L. Her thyroid-stimulating hormone (TSH) was 2.24 mIU/L. Contrast-enhanced computed tomography of the chest, abdomen, and pelvis revealed peripherally enhancing well-defined local lesion in the body of the pancreas measuring 3.2 × 2.7 × 2.8 cm with a small cystic component without any evidence of metastasis or infiltration (Figure 1). Colonoscopy revealed only mild colitis in the rectum and descending colon. Histology was normal.

**Table 1:** Laboratory values WBC: white blood cells, ALT: alanine aminotransferase, AST: aspartate aminotransferase, TSH: thyroid-stimulating hormone, LDH: lactate dehydrogenase, -: investigation not repeated

	On admission	After giving octreotide	After surgery	Normal values
FBC				
WBC (10^9^/L)	10.13	8.43	9.52	4–11 (10^9^/L)
Neutrophils	6.01	5.04	7.19	2–7 (10^9^/L)
lymphocytes	3.30	2.64	1.79	1–3 (10^9^/L)
Hemoglobin	12.1	11.3	12.2	12–15 (g/dl)
Platelets	345,000	263,000	275,000	150–410 (10^9^/L)
Electrolytes				
Serum sodium	141	142.6	138	136–145 (millimole/L)
Potassium	2.0	4.32	4.2	3.5–5.1 (millimole/L)
Calcium	2.3	-	-	2.10–2.54 (millimole/L)
Magnesium	0.9	-	-	0.66–0.95 (millimole/L)
Phosphorus	0.9	-	-	0.81–1.45(millimole/L)
Urinary sodium	80	-	-	<20 (millimole/L)
Urinary potassium	18.3	-	-	<20 (millimole/L)
Stool sodium	83	-	-	(millimole/L)
Stool potassium	56.17	-	-	(millimole/L)
Blood urea	2.1	2.8	3.6	2.5–6.4 (millimole/L)
Serum creatinine	64	68	56	49–90 (micromole/L)
Serum osmolality	281	-	-	275–295 (milliosmole/kg)
Urine osmolality	382	-	-	50–1,200 (milliosmole/kg)
Stool osmolar gap	12	-	-	-
Serum bicarbonate	12.1	-	-	22–28 (millimole/kg)
Serum ALT (U/L)	82	36.1	47	16–63 (units/L)
Serum AST (U/L)	46	39. 1	31	15–37 (units/L)
Stool full report				
WBC	Nil	-	-	-
RBC	Nil	-	-	-
Amoeba	Nil	-	-	-
Ova	Nil	-	-	-
Cyst	Nil	-	-	-
Blood	Nil	-		-
Stool culture	No growth	-	-	-
Stool Calprotectin	Normal	-	-	-
TSH	2.23	-	-	0.465–4.68 (milli-international units/L)
LDH	197	-	-	140–280 (units/L)
HIV/VDRL	Negative	-	-	-
Serum amylase	69	-	36	40–140 (units/L)

**Figure 2:**
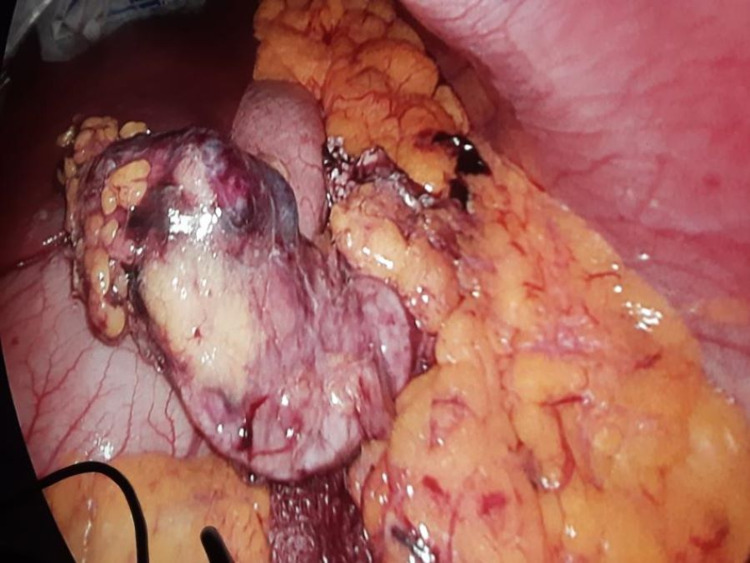
Laparoscopic view of the tumor attached to the body of the pancreas

**Figure 3:**
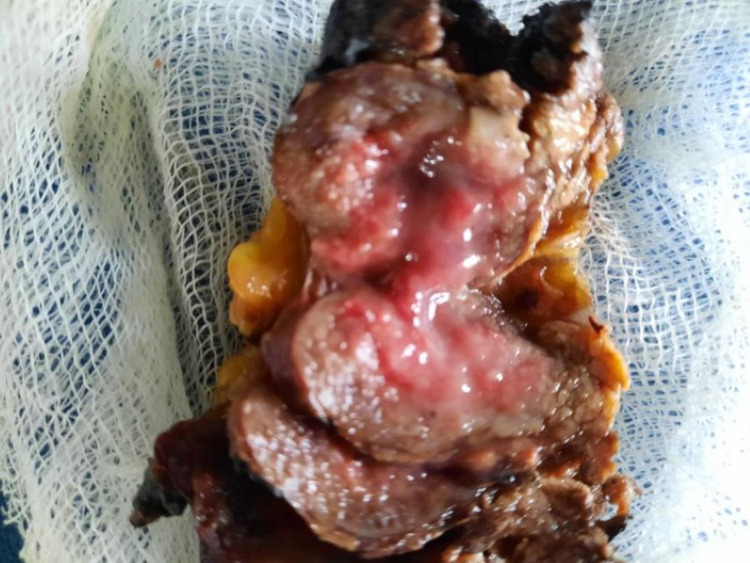
Cut surface of the pancreatic neuroendocrine tumor

**Figure 4:**
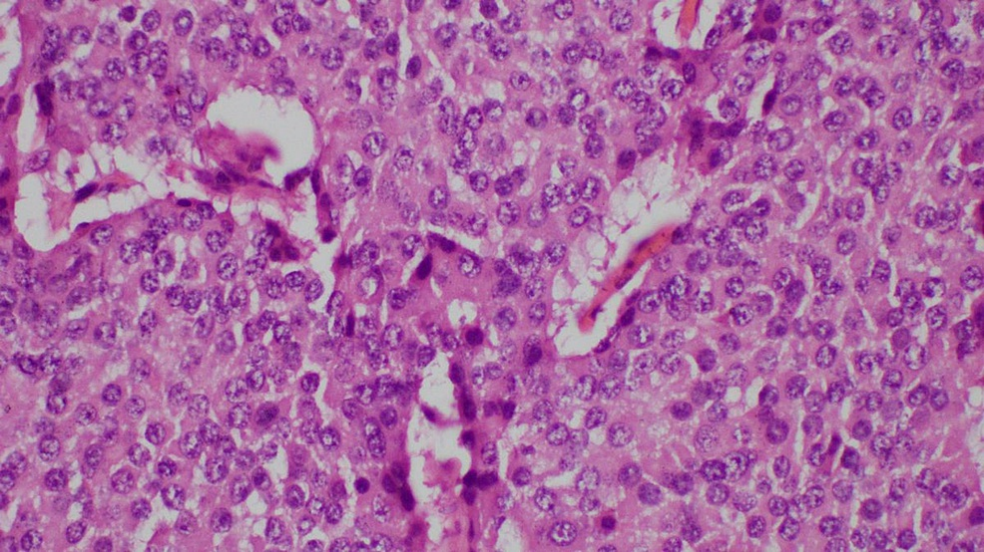
Histology of the specimen after hematoxylin and eosin staining with ×400 magnification showing partly encapsulated lesion composed of nests and trabeculae of tumor cells; cells have monomorphic round nuclei, and the cytoplasm is eosinophilic and moderate in amount; scanty mitosis and no necrosis were seen

**Figure 5:**
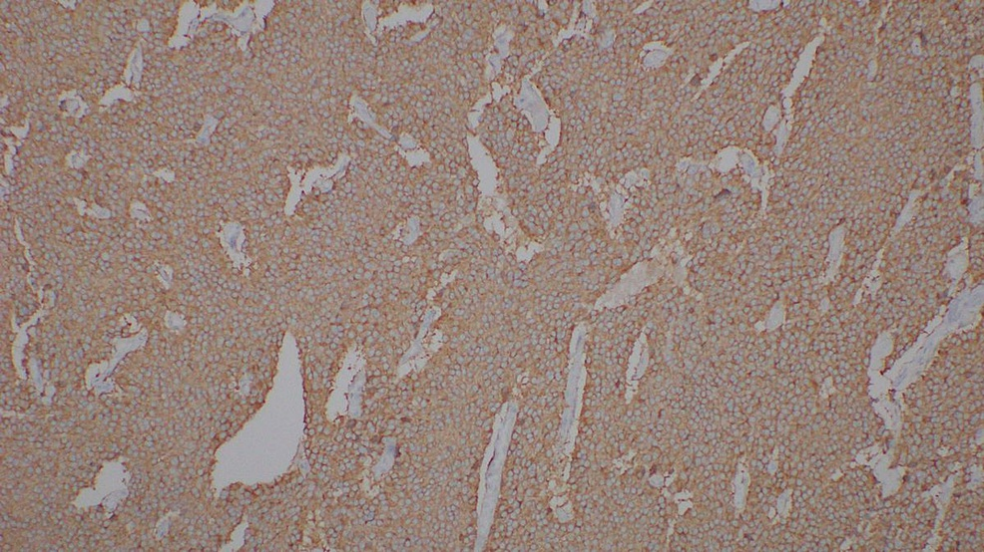
Tumor cells show diffuse and strong cytoplasmic positivity with synaptophysin, a marker glycoprotein for neuroendocrine cells

Vasoactive intestinal peptide-secreting pancreatic neuroendocrine tumor was made as a possible diagnosis after imaging and blood investigations. Serum VIP levels and somatostatin receptor scintigraphy were not done due to nonavailability. Octreotide 50 µg S/C twice daily was initiated as the therapy. After four days, she was able to maintain her potassium above 4.0 mmol/L with only oral replacement, and oral potassium was reduced and stopped completely during the course. She underwent laparoscopic distal pancreatectomy and removal of the tumor from the pancreatic body (Figure 2). The biopsy and immune histochemistry features were suggestive of a histological diagnosis of well-differentiated pancreatic neuroendocrine tumor (G1/G2) (Figures 3, 4) and strong cytoplasmic positivity with synaptophysin (Figure 5).

## Discussion

VIPoma is a rare pancreatic neuroendocrine tumor causing chronic diarrhea. Most of them are malignant, and only 10% are benign [2]. They commonly occur in the tail and body of the pancreas. The typical age of presentation is the fourth and fifth decades [2]. Patients without metastatic disease have a higher five-year survival rate (>90%) compared with patients with metastatic disease (60%).

VIP is a 28-amino acid neuropeptide that causes large voluminous watery diarrhea by its vasodilator effect on intestinal smooth muscle and stimulating the production of CAMP in enterocytes, which induces the secretion of water and electrolytes, especially potassium and bicarbonate, along with water into the intestinal lumen [3].

The differential diagnosis of large voluminous fasting diarrhea includes Zollinger-Ellison syndrome, laxative abuse, and rarely human immunodeficiency virus (HIV). VIPoma can be differentiated from Zollinger-Ellison syndrome by the absence of gastric hypersecretion and moderate loss of potassium in stool and metabolic acidosis [4]. Serum gastrin levels were not done due to unavailability.

Other investigations include serum pancreatic polypeptide and chromogranin A. Functional PET imaging such as 68-Ga DOTA fusion PET/CT scans have a sensitivity of about 97% compared with CT scans [2]. The World Health Organization (WHO) classified these neuroendocrine tumors according to mitotic rate and Ki67 index. It includes three grades (G1, G2, and G3). G1 means Ki67 less than 3% (low grade), G2 means 3%-20% (intermediate), and G3 means more than 20% (high grade) [5]. Our patient’s Ki67 is 3%, so it was a well-differentiated intermediate neuroendocrine tumor with no metastasis.

The definitive treatment for VIPoma is surgery; it improves disease-free survival in both primary and metastatic diseases. Treatment with somatostatin analog octreotide quickly resolves diarrhea. The main action of the drug is the direct inhibitory effect on the production of hormones from the tumor [3]. Inoperable tumors are managed by palliation therapy, including cytoreductive surgery for symptom control along with biologics and chemotherapy. Although novel agents such as sunitinib were proven to be effective in controlling the symptoms of VIPoma, we did not try them in our case. The poor prognosis of VIPoma is associated with histological grade, staging, and the presence of metastasis [6].

## Conclusions

Although VIPoma is rare, when a patient presents with chronic secretory diarrhea with persistent hypokalemia and metabolic acidosis, a diagnosis of VIPoma should be considered after excluding other possible causes. Early diagnosis prevents progression and complications. We made the diagnosis based on clinical and histological basis as the measurement of serum VIP level is not available in our country. Currently, the patient is symptom-free, and she is on regular six-month follow-up. The absence of metastasis, younger age, and complete resection of the tumor contributed to her good prognosis. We consider this case atypical presentation because of the absence of metastasis since most of the reported cases illustrate VIPoma that usually metastasizes to the liver or lung at the time of diagnosis.

## References

[1] Verner JV, Morrison AB (1958). Islet cell tumor and a syndrome of refractory watery diarrhea and hypokalemia. Am J Med.

[2] Shankar N, Linzay C, Rowe K (2020). Vasoactive intestinal peptide-oma causing refractory diarrhea in a young woman. Proc (Bayl Univ Med Cent).

[3] Natanzi N, Amini M, Yamini D, Nielsen S, Ram R (2009). Vasoactive intestinal peptide tumor. Sch Res Exch.

[4] Taheri S, Ghatei MA, Bloom SR (2010). Gastrointestinal hormones and tumor syndromes. Endocrinology.

[5] Nagtegaal ID, Odze RD, Klimstra D (2020). The 2019 WHO classification of tumours of the digestive system. Histopathology.

[6] Sánchez-Salazar SM, Torres-Alzate S, Muñoz-Cortés VM, Builes-Barrera CA, Gutiérrez-Montoya JI, Román-González A (2021). VIPoma: a rare cause of diarrhea. A case report. Rev Fac Med.

